# Estimation of Eye Closure Degree Using EEG Sensors and Its Application in Driver Drowsiness Detection

**DOI:** 10.3390/s140917491

**Published:** 2014-09-18

**Authors:** Gang Li, Wan-Young Chung

**Affiliations:** Department of Electronic Engineering, Pukyong National University, Busan 608-737, Korea; E-Mail: ligang@pknu.ac.kr

**Keywords:** driver drowsiness detection, eyelid closure degree, EEG, linear regression

## Abstract

Currently, driver drowsiness detectors using video based technology is being widely studied. Eyelid closure degree (ECD) is the main measure of the video-based methods, however, drawbacks such as brightness limitations and practical hurdles such as distraction of the drivers limits its success. This study presents a way to compute the ECD using EEG sensors instead of video-based methods. The premise is that the ECD exhibits a linear relationship with changes of the occipital EEG. A total of 30 subjects are included in this study: ten of them participated in a simple proof-of-concept experiment to verify the linear relationship between ECD and EEG, and then twenty participated in a monotonous highway driving experiment in a driving simulator environment to test the robustness of the linear relationship in real-life applications. Taking the video-based method as a reference, the Alpha power percentage from the O2 channel is found to be the best input feature for linear regression estimation of the ECD. The best overall squared correlation coefficient (SCC, denoted by r^2^) and mean squared error (MSE) validated by linear support vector regression model and leave one subject out method is r^2^ = 0.930 and MSE = 0.013. The proposed linear EEG-ECD model can achieve 87.5% and 70.0% accuracy for male and female subjects, respectively, for a driver drowsiness application, percentage eyelid closure over the pupil over time (PERCLOS). This new ECD estimation method not only addresses the video-based method drawbacks, but also makes ECD estimation more computationally efficient and easier to implement in EEG sensors in a real time way.

## 1. Introduction

Driver drowsiness is one of the major causes of mortality in traffic accidents worldwide. Among the members of Organization for Economic Co-operation and Development (OECD), South Korea has the highest car accident mortality rate [1]. From 2010 to 2013, 1223 people died in highway traffic accidents in Korea, and 31% of them died in accidents related to driver drowsiness [2,3]. Driver drowsiness is mainly caused by long duration monotonous driving, and is characterized by remarkable behavioral changes, including variation in the pupil size, blinking of the eyes and various body movements [4]. One of the milestones in the monitoring of driver drowsiness is the usage of percentage eyelid closure over the pupil over time (PERCLOS) to give a warning if driving whilst drowsy is determined. It has previously been verified that PERCLOS is the most reliable and valid in-vehicle drowsiness detection technology [5]. PERCLOS is a video-based driver drowsiness monitoring technology. It assesses drowsiness by measuring slow eyelid closure and estimating the proportion of time for which the eyes are closed over specified time intervals. According to the documents published by the US Federal Highway Administration (FHWA) [6,7], the measurement of high sensitive PERCLOS is given by Equation (1), where ECD refers to Eyelid Closure Degree:

(1) $$\text{PERCLOS}=\frac{\text{Time}(\text{ECD}\geq 80\%)}{1\min}\times 100\%$$

Conventionally, the calculation of the ECD is based on image processing, which requires proper brightness and a stable face-to-camera distance. There are however some limitations, for example, the ECD cannot be properly determined when drivers work during the night or when drivers wear glasses in the daytime. In addition to this, video based systems need to overcome practical hurdles, such as causing distraction of the driver. This point can be indirectly verified by the growing number of traffic accidents caused by the use of in-vehicle Digital Multimedia Broadcasting (DMB) and smartphones in Korea [1], where drivers who read, text and watch the DMB or smartphone are driving whilst distracted. Also, the high-computational load is another disadvantage of the video-based method. For example, in previous study [8], we developed a video-based PERCLOS system using an Android smartphone's built-in camera (Galaxy SIII, Samsung, Korea). The minimum preview size for its built-in camera is 320 × 240, which needs at least 150 KB buffer size. Such a high computational load is not suitable for the low-power requirement in field application. Thus, the success of traditional video- based measurement is very limited.

EEG is a non-invasive brain activity measurement method. Based on different frequencies, EEG signals can be categorized into five specific bands: Delta (δ, below 4 Hz), Theta (*θ*, 4–7 Hz), Alpha (*α*, 8–12 Hz), Beta (*β*, 13–30 Hz) and Gamma (*γ*, above 30 Hz). The occipital EEG has a direct relationship to ECD since the visual cortex, which is responsible for processing visual information, is located in the occipital region of the brain. The well-known sleep recording standard today, the American Academy of Sleep Medicine's (AASM, 2007 [9]), just determines Stage W (wake) using following occipital eye closure (EC)/open (EO) related EEG features: In stage W, the majority of individuals with eyes closed will demonstrate an *α* rhythm, whilst the EEG pattern when the eyes are open consists of low amplitude activity without the rhythmicity of an *α* rhythm. Other studies about the EEG and ECD are more interesting. For example, Mulholland and Evans [10] showed that people were able to decrease the *α* wave ‘at will’ by visually fixating, and increase it by blurring the image. Craig *et al.* [11] developed an algorithm for the disabled to control electrical devices using the EC-related increase and EO-related decrease in amplitude of *α* wave. Also, Ianov *et al.* [12] tested subjects in a dark room with a lamp in front of them. Strong *α* signals could be obtained when the light was turned off, whereas a weak signal was recorded when the light was on.

These discoveries demonstrate the interesting interactions between EEG and ECD, however most of them mainly focused on the EC/EO applications and did not quantify the ECD changes and thus could not accurately determine on the nature of the relationship between EEG and ECD. This study firstly quantified the ECD changes using a self-developed device and did a proof-of-concept experiment to assess whether there is a linear relationship between occipital EEG and ECD. Then, a monotonous driving simulation experiment was carried out to assess the robustness of the relationship in a real-life application. Experimental results indicate that a linear relationship between EEG and ECD does exist. Therefore, a linear support vector regression model (SVR) is further proposed to predict the driver's ECD using EEG power spectrum features and compared with two non-linear SVR models: (1) SVR with radial basis function (RBF) kernel and (2) SVR with polynomial kernel. This new ECD estimation method not only addresses the video-based drawbacks, but also lays the foundation for the development of a driver drowsiness detector which uses EEG alone but is characterized by multi-channel data fusion methods: occipital EEG for estimating ECD, frontal and central EEG for measuring the conventional fatigue features (e.g., *θ/β*, *θ/(α+β)*,*(θ+α)/β* and *(θ+α)/(α+β)* [13]).

## 2. A Proof-of-Concept Experiment

A proof-of-concept experiment is firstly carried out to assess whether there is a linear relationship between occipital EEG and ECD. The linear relationship is quantified using a simple linear regression model.

### 2.1. Subjects and Data Collection

In order to ensure the good contact quality of electrodes and scalp, ten male subjects (age 26.1 ± 1.97 years) who had shorter hair participated in the pilot experiment. None of the subjects reported any ocular impairment and did not drink tea or anything containing caffeine before the experiment. During the experimental procedure, each subject was instructed to do eyelid movements as follows: full open (FO), slight closure (SC), half closure (HC), almost closure (AC) and full closure (FC), in order to obtain linearly growing ECD values. Initially, we intended to quantify the eyelid movement using fixed values, such as 20% ECD for SC, 50% for HC and 80% for AC, however, this was too ideal to have them performed by the subjects. Thus, in this study, the five eyelid movements were controlled by the subjects themselves as long as each ECD would correctly fall into the five pre-defined groups, as shown in Table 1, following this the ECD was increasing, but not exactly in a linear increase.

**Figure 1.**
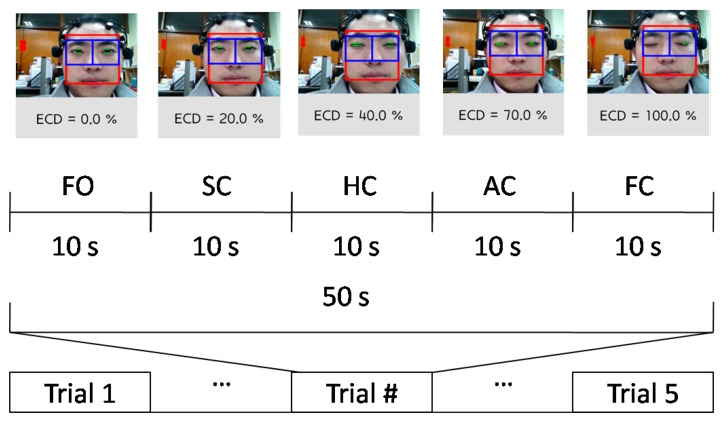
Trial structure adopted in the experimental protocol.

**Figure 2.**
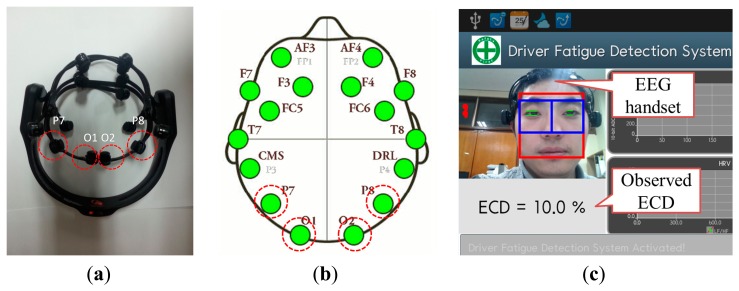
(**a**) EEG headset and occipital electrode positions, according to the 10–20 systems, of the Emotiv EPOC device used for EEG acquisition. (**b**) The contact quality of electrodes and scalp is good (green color). (**c**) Experimental configuration: quantitatively measure the ECD using a video-based method meanwhile testing the EEG.

According to common sense, 10 s is an accepted longer duration for subjects to maintain a certain ECD group without any eye blinks. Therefore, in this study, each ECD group was maintained for 10 s duration without any eye blinks. Subjects were asked to implement the ECD groups orderly from FO to FC. During each ECD group, subjects were required to remain as still as possible, in order to reduce muscular artifacts. The five ECD groups correspond to one trial. Each subject was asked to repeat the trial five times, in order to avoid accidental EEG signals. The structure of each trial is shown in Figure 1. EEG recordings were conducted using the Emotiv EPOC 14-channel EEG wireless recording headset (Emotiv Systems, Inc., Hong Kong). The electrodes were placed according to the international 10–20 system and included active electrodes at occipital region: P7, O1, O2 and P8 (Figure 2a,b, red circles with dotted line).

The contact quality of electrodes and scalp is color coded in the software that pairs with the commercialized EEG device (Black: no signal; Red: very poor signal; Orange: poor signal; Yellow: fair signal; Green: good signal). EEG data is band-pass filtered in the range of 0.2–45 Hz using hardware filters. Two digital notch filters at 50 and 60 Hz are further applied, and the output data are sampled at 128 Hz. The portability and easy-to-wear characteristics of the recording device and high resolution (16-bit ADC) are the reasons for its use in this study. For reference, the video-based ECD values were obtained at the same time with EEG recordings as shown in Figure 2c. The video-based device was developed by our previous work [14], which is a smartphone application using the built-in camera to recognize the face first and then perform ECD calculations. The ECD sampling rate was 7 Hz. All subjects sat comfortably on a chair in a laboratory environment under daylight lamp conditions.

### 2.2. Power Spectrum Analysis

In order to reduce the individual differences, EEG power percentages (*θ*, *α* and *β* power percentages) instead of the absolute EEG power values are calculated from the EEG raw data. EEG power is calculated as the sum of the squared FFT magnitude of the EEG signal using a 10 s Hamming window. Then, The power percentage (Per) is calculated as the result of dividing the FFT power of one EEG band by the sum of the FFT power of all three EEG bands (Equation (2), where, *z_i_* = {*θ*, *α*, *β*}). The FFT analysis was implemented using the Complexity software (Ver. 2.82, Laxtha, Daejeon, Korea). Before power spectrum analysis, all EEG signals are filtered using basic FIR filter with 4–30 Hz bandwidth in order to filter out delta and gamma wave. Delta wave (0–4 Hz) and Gamma wave (30–100 Hz) are mainly related to deep sleep and arousal effects, respectively [9,15], which is beyond our study topic.

(2) $$\text{Per}(z_{i})=\frac{\text{Power}(z_{i})}{\overset{3}{\underset{i=1}{\text{∑}}}\text{Power}(z_{i})}\times 100\%$$

### 2.3. Simple Linear Regression Model

Assuming that the ECD and the extracted EEG feature *f* has a linear relationship, a simple linear regression model can be described by *ECD_i_* = *α* + β* *f_i_* + *ε_i_*, where *ε_i_* is assumed to be the random zero mean noise, *α* is the intercept and *β* is the slope of the line which specifies how much the contributes to the *ECD_i_*. Since *ε_i_* is a random factor, we cannot directly determine *ECD_i_*. Therefore, in order to estimate the ECD (*eECD*) for a given *f_i_*, *α̂* and *β̂* are needed to be estimated by using the least squares prediction equation *eECD_i_* = *α̂* + β̂* f_i_. Given *n* observation pairs, {(*ECD_i,_*, *f_i_*)}, where *i* = 1,…,*n*, it is possible to find the values of *α̂* and *β̂* that minimize ${\overset{n}{\underset{i=1}{\text{∑}}}\left(ECD_{i}-eECD_{i}\right)}^{2}$, so we have:

(3) $$\alpha =\bar{ECD}-\hat{\beta }*\bar{f}$$

(4) $$\hat{\beta }=\frac{Cov(f,ECD)}{Var(f)}$$

where, *Cov*(*f*, ECD) is the covariance of feature *f* and observed ECD and, Var (*f*) is the variance of feature *f*.

### 2.4. Results

**Figure 3.**
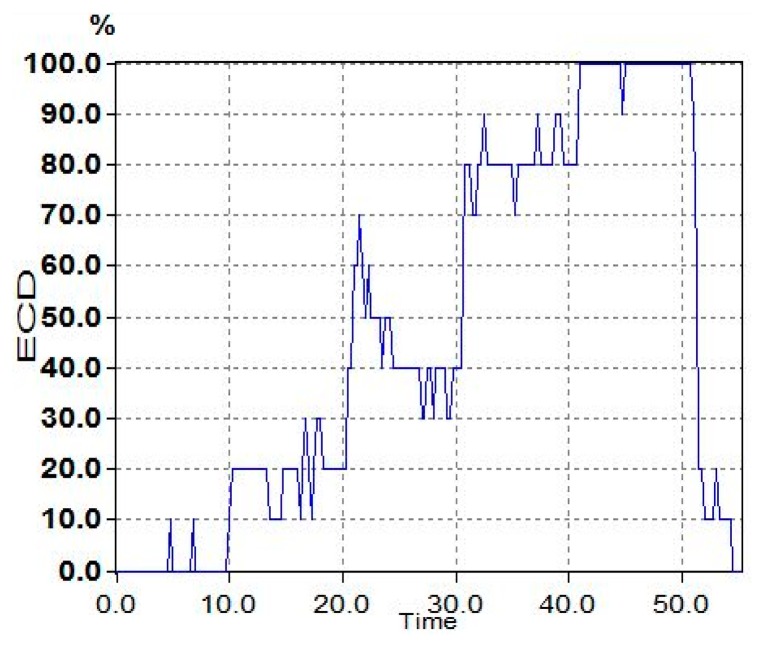
The typical signals for one trial ECD plots.

**Figure 4.**
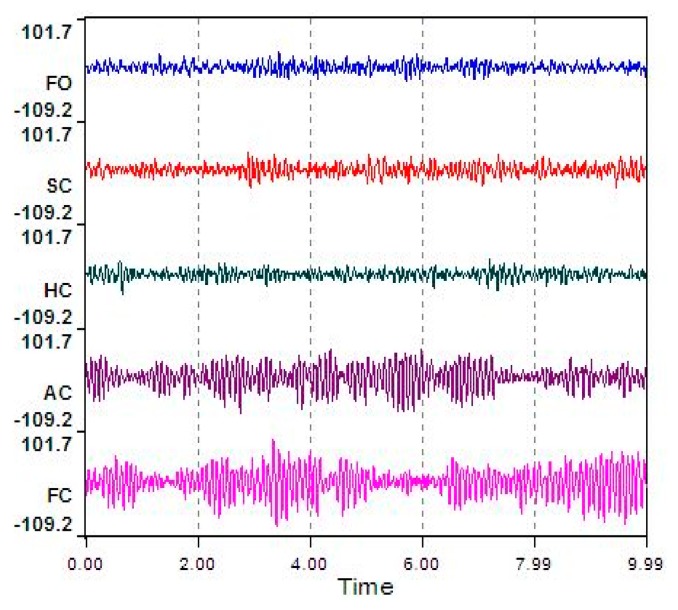
The typical signals for one trial EEG signals.

**Figure 5.**
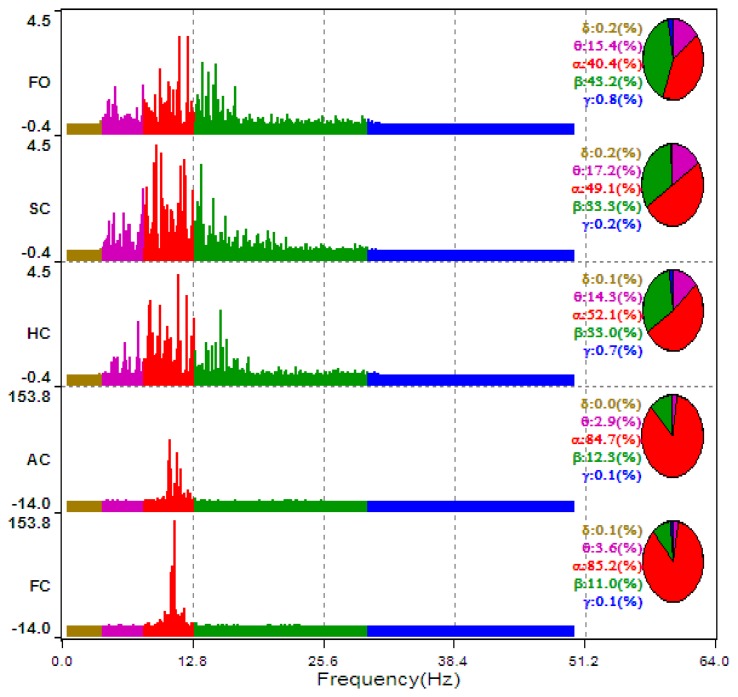
The typical changes of power percentage for one trial EEG signals as the increase of ECD.

The typical ECD plots and occipital EEG recordings (at electrode O2) for one trial (from a representative subject) are shown in Figures 3 and 4. Visually, the difference in the EEG signals among the five ECD groups indicates a linearly growing low-frequency component. FFT analysis shows that this component is located in EEG *α* band (red color in Figure 5).

**Figure 6.**
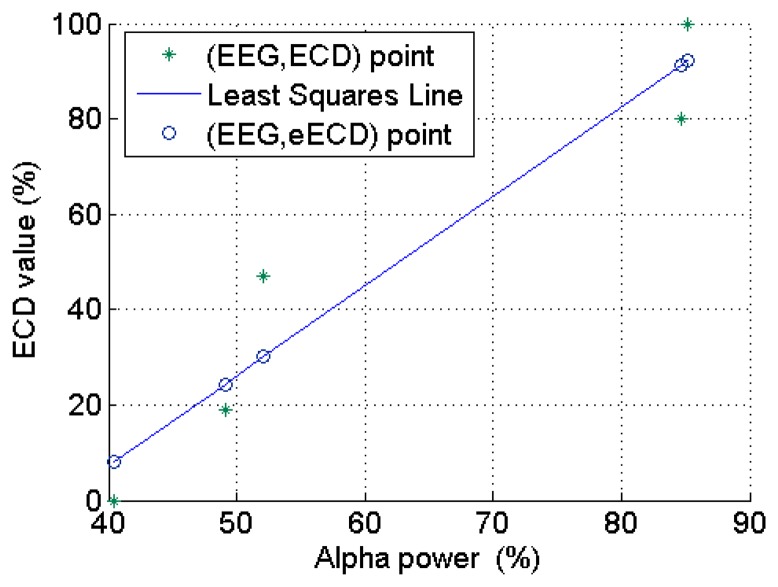
The simple linear regression model used to quantify the linear relationship between ECD and EEG.

The averaged ECD values for each ECD group in Figure 3 and extracted EEG *α* power percentage in Figure 5 are further summarized in Table 2. Based on these results, a simple linear regression model is used to quantify the linear relationship between this representative subject's ECD and EEG (as shown in Figure 6) with slope *β^* (= 1.878), intercept *α̂* (= −67.84) and *R^2^* = 0.917. *R^2^* is a measure of how goodness of linear fit. If *R^2^* = 1.00, that means perfect linear relationship.

**Figure 7.**
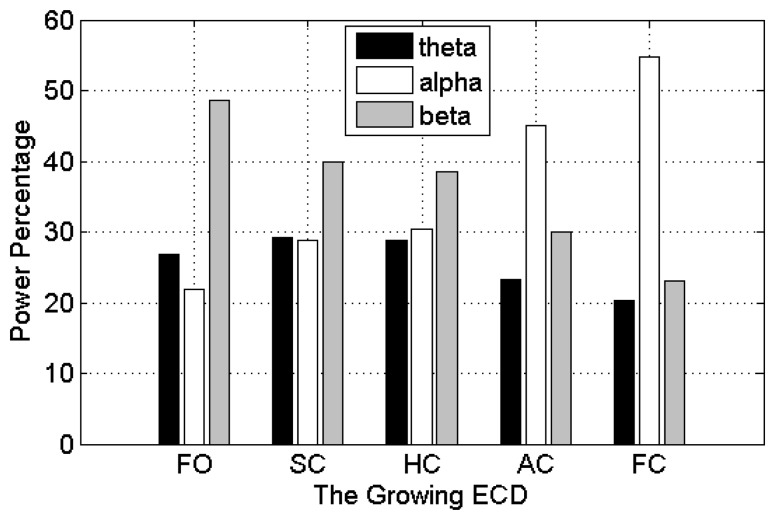
The EEG power spectrum features with the growth of ECD.

The averaged *θ*, *α* and *β* power percentages over the four occipital channels over the 10 subjects were used to assess whether these EEG features show linear relationship with ECD. As can be seen in Figure 7, α wave presents a positive and linear trend with the growth of ECD, while *β* wave shows a linear relationship with negative trend. However, *θ* wave does not show linear trend. The *θ* wave increases from 27% at FO to 30% at SC and maintains about the same value at HC, and then has a significant decrease at AC until to 20% at FC stage. These results show that occipital EEG, particularly the *α* wave, indeed exhibits a linear relationship with ECD when subjects did not experience much cognitive loads.

## 3. Driving Simulation Experiment

In order to assess the robustness of this linear relationship in a real-life application, a 2 h monotonous driving experiment, which involved the participants driving on a highway, was implemented in a driving simulation environment. During the two hours, subjects experienced various cognitive loads in a monotonous driving level, such as keeping or changing lanes for avoiding car collisions. Also, subjects experienced different daylight conditions in this driving game, such as driving during the daytime or at nighttime.

### 3.1. Experimental Setup

**Figure 8.**
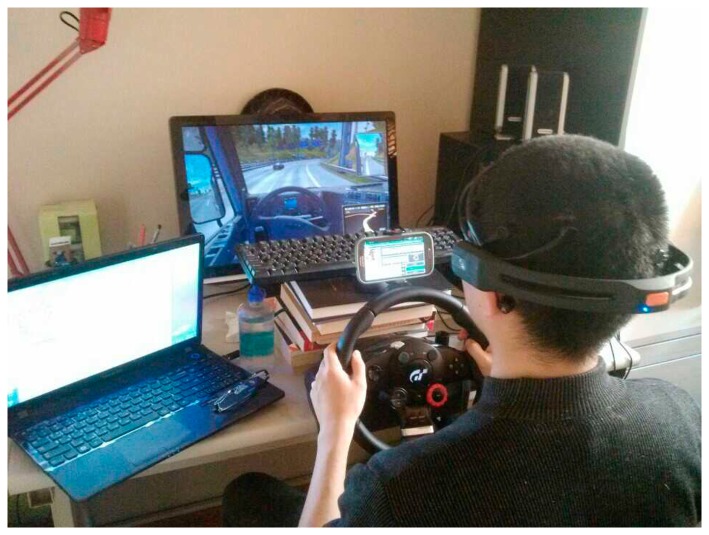
Example of the experimental setup for monotonous driving.

According to our previous experiences [8,14,16,17] and related studies [18,19], 1 h monotonous driving after lunch (usually 1:00 pm∼2:30 pm) could make most of subjects feel drowsy. In this study, in order to make subjects tired enough and get FC data as much as possible, we extended the experiment to two hours. Figure 8 shows the driving simulation environment, which consists of a commercial truck driving game (Euro Truck Simulator 2), a Logitech^®^ steering wheel, accelerator and brake pedals. The smartphone, which is responsible for monitoring subjects' ECD values, is placed behind the steering wheel.

### 3.2. Subjects and Data Collection

A total of twenty subjects (age 25.6 ± 2.17 years) who already had a driving license participated in this driving experiment. Half of them (five males (subjects #1∼5) and five females (subject #11∼15)) participated in the daytime driving experiments. The remainder did the nighttime driving experiments. None of them reported any ocular impairment. On the day of experiment, all subjects were not allowed to drink tea or anything containing caffeine. Also, no soporific medicine, such as medicines for treating a cold, was allowed. Before the experiment, each subject was given 10 min to become familiar with the operation of the driving simulator. The EEG and ECD collection procedure were slightly different than in the aforementioned proof-of-concept experiment. Here, we projected the smartphone screen onto a PC using the Mobizen software (Mobizen. Inc, Bucheon, Korea) and then recorded the smartphone screen and real time EEG waveform in the PC using the Bandicam software (Bandicam.Inc, Seoul, Korea), in order to record the timing of artifacts and later extract target segments of interest.

### 3.3. Pre-Processing

Firstly, we directly rejected artifacts including chin EMG, yawn, and body movements according to the recorded video evidence. Then, we filtered eye blinks in the EEG data using the Independent Component Analysis (ICA) in the EEGLAB Toolbox (Ver. 7.1.3.13b) which is developed by the Swartz Center for Computational Neuroscience, (University of California San Diego) [20], while the removal of eye blinks in ECD was by a self-developed algorithm whose main idea is to find the threshold ECD values of eye blinks (90%–100%) within 400 ms (the average duration of a single eye blink is less than 400 ms, according to the Harvard University database of useful biological numbers [21]) and then replace the high ECD values with their previous ECD values.

After artifacts were removed, we extracted the EEG datasets which correspond to the five ECD groups (FO∼FC). However, unlike the same ECD durations (10 s) in the previous proof-of-concept study, each ECD group, in this 2 h real-life application, did not appear continuously but rather consisted of several discrete segments with different durations. For example, one subject may experience several FO segments at different times in the 2 h experiment and these discrete FO segments form the FO group. Most of the subjects easily maintained the FO segments for a few minutes; however, most of them could only maintain the FC segments for few seconds, in order to drive continuously. Therefore, it is not reasonable at this time to select each ECD group and corresponding EEG datasets with the same 10 s window. We selected five segments with the longest durations in their own groups as the representatives of the five ECD groups. Then, we extracted the EEG datasets which correspond to the five representative ECD segments. If the duration is longer than 10 s, we only selected the first 10 s. Finally, all EEG segments in the four occipital channels (P7, O1, O2 and P8) are filtered using basic FIR filter with 4–30 Hz bandwidth for further study.

### 3.4. Extracted EEG Features

For a more comprehensive analysis, two time features, Root mean square (RMS) and Shannon entropy (SE), were newly added at this time. Traditionally, a time domain EEG analysis can be accomplished by examining how the voltage changes over time, for example, by examining the mean and variance of the sampled waveform. Since the EEG can be considered a *zero mean* Gaussian random process (the voltage of which is positive as often as it is negative) [22], the RMS, which utilizes absolute values, is an appropriate method to calculate the mean EEG amplitude. The use of RMS values is not uncommon in the analysis of physiological signals. For example, an EMG also has a mean voltage of zero over time and uses the RMS to compute its mean amplitude [23,24]. SE was first introduced by Shannon [25], and can be viewed as a measure of the amount of information [22], particularly as a measure of the transient or time varying changes [26,27]. Thus, in order to get information on how much the EEG differs with the transient ECD changes, the SE was also studied here. A brief description of the time features is as follows:

#### 3.4.1. RMS

The RMS is given by Equation (5), where *s_i_* (*i =* 1,…,*n*) is the sampled EEG data (digitalized amplitude value) and *n* is the number of sample data points:

(5) $$RMS=\sqrt{\frac{\sum_{i=1}^{n}s_{i}^{2}}{n}}$$

#### 3.4.2. SE

The SE is defined as in Equation (6), where *s_i_* (*i =* 1,…,*n*) is the sampled EEG data (digitalized amplitude value), *n* is the number of sample data points and *p*(*x_i_*) is the probability that the amplitude value *s_i_* occurs anywhere in the signal. The *p*(*s_i_*) is estimated by a histogram method where the amplitude range of the EEG signal is linearly divided into k bins. In this study, b is chosen as 10 and built-in Matlab function *hist*() is used to get the histogram of EEG samples, where the *k* = 10 (default):

(6) $$SE=\frac{-\sum_{i=1}^{n}p(s_{i})\times \log_{b}p(s_{i})}{\log_{b}k}$$

Therefore, a set of five features including time- and frequency domain features were extracted from the EEG signals. The summary of the features used is given in Table 3. The RMS, SH and FFT (Power percentage, Per) are computed based on each ECD group and four occipital channels. Therefore, twenty feature values were obtained from each subject. Each feature (*f*) can be denoted as *f = u*(*x,y,z*), where *f* ∈ {RMS, SH, Per}, x denotes the ECD type ∈ {FO, SC, HC, AC, FC}, y denotes the EEG channel ∈ {P7, O1, O2, P8} and z denotes the EEG band ∈ {*θ, α, β*}.

### 3.5. Experimental Results

#### 3.5.1. Overview

**Figure. 9.**
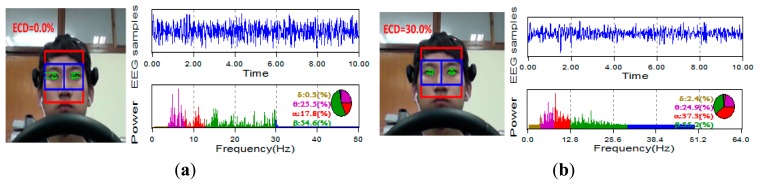

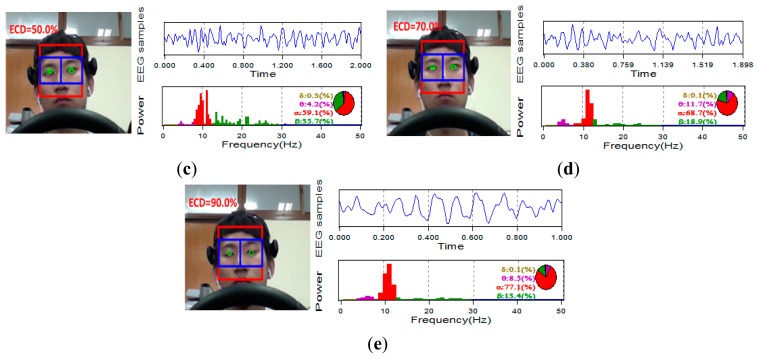
The typical EEG recordings and their time-frequency analysis that vary with the five ECD groups. (**a**) FO group (**b**) SC group (**c**) HC group (**d**) AC group (**e**) FC group.

All subjects experienced drowsy driving symptoms (e.g., yawns), according to our recorded video. Fifteen out of them experienced all ECD groups (FO∼FC). As mentioned in Section 3.3 Pre-processing, we selected a total of 90 ECD segments from the 20 subjects. More information can be found in Table 4. The typical EEG recordings that are from subject #1's O2 position and the EEG power spectra that vary with the five ECD segments are illustrated in Figure 9, where we can see clearly that the *β* power which is in the range of 12∼30 Hz is gradually decayed as the ECD increases, while the *α* power, which is in the 8–12 Hz range, is increasing as the ECD increases.

**Figure 10.**
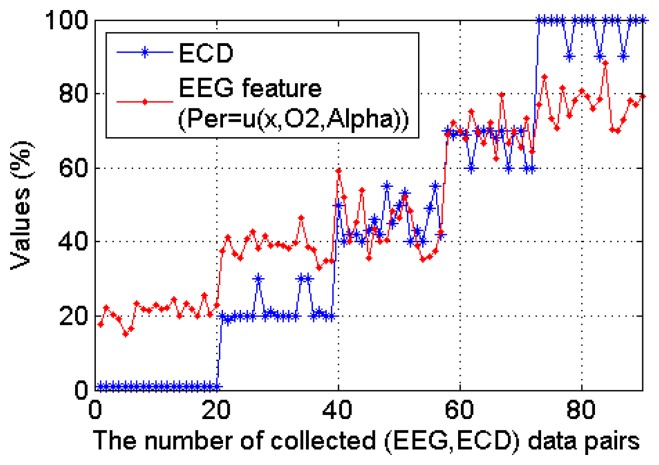
The linear relationship between ECD and a representative EEG feature which were collected from 20 subjects from a real-life experiment environment (monotonous driving experiment).

**Figure 11.**
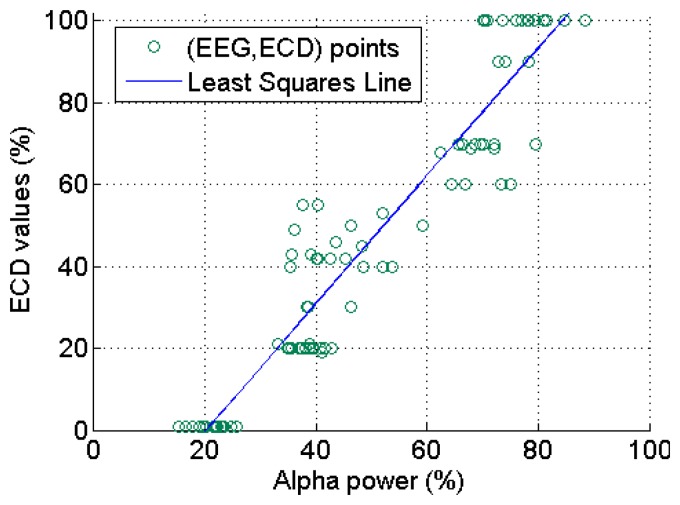
The simple linear regression model with slope *β^* (= 1.560) and intercept *α̂* (= −31.370) from a representative subject (*R^2^* = 0.904).

All the collected 90 ECD and EEG (*α* power percentage at O2 channel) data pairs from the 20 subjects are plotted in Figure 10, where we can see clearly that this occipital EEG feature exhibits a positive linear relationship with the ECD. As shown in Figure 11, a simple linear regression model quantifies this linear relationship with the squared correlation coefficient R^2^ = 0.904.

#### 3.5.2. Relationship of EEG Features with ECD

**Figure 12.**
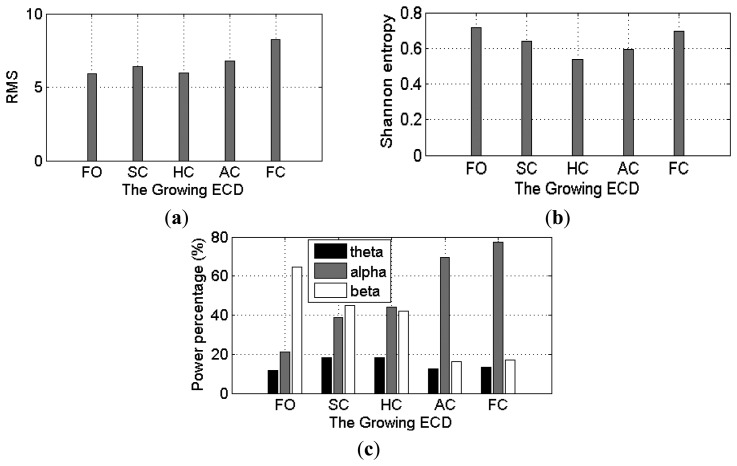
The time- (**a, b**) and frequency-domain (**c**) EEG features with a growth of the ECD.

The averaged RMS, Shannon entropy, theta, α and β power percentages over the four channels for the 20 subjects were used to assess whether these EEG features exhibit a linear relationship with the ECD. The overall results are shown in Figure 12, where we can see clearly that with an increase of the ECD, the α power percentages and the fluctuations of the RMS values exhibit a tendency to increase, whilst the β power percentages show a decreasing trend (except for the slight increase at the FC stage). Shannon entropy exhibits a “U” shape tendency, whilst theta power percentages show an approximately inverted U-shaped tendency. The θ wave increases from 12% at FO to 18% at SC and maintains about the same value at HC, and then has a decrease at AC until to another slight increase at FC stage (14%).The authors are unable to provide a full physiological reasoning behind the Shannon entropy and theta activity at this time; however the other results are reasonable and what would be expected because they can be thought of as the interaction between the EEG and visual cortex. More details about the EEG and visual cortex can be found in Section 6.1.

#### 3.5.3. Feature Selection for Building ECD Prediction Model

**Figure 13.**
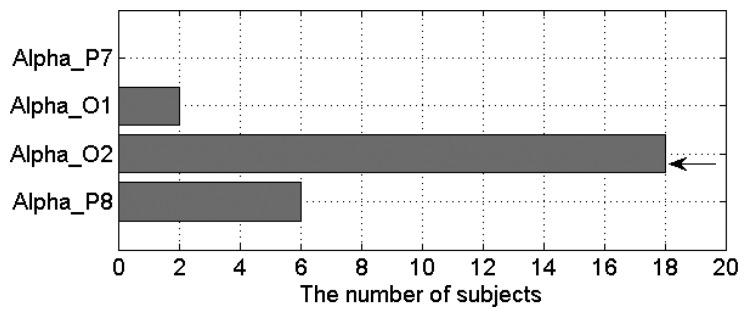
The bar graphs show the number of subjects that show positive linear relationship between ECD and α power percentage in each occipital channel. The arrow indicates the best occipital channel. The *Y*-axis indicates the EEG features (Alpha_P7—*Per = u*(*x, P7, α*)). The *X*-axis indicates the number of subjects that exhibit an increasing α power percentage as the ECD increases.

Based on the results shown in Figure 12, we found that *α* power percentage outperforms other EEG features. To further select the best occipital channel, the number of subjects that show positive linear relationship between ECD and *α* power percentage was investigated for each occipital channel (as shown in Figure 13). The single-best EEG feature is found to be the *α* power percentage from the O2 channel (*Per = u*(*x, O*2, *α*)), which of 18 out of 20 subjects (nine males and nine females) exhibit an increasing *α* power percentage as the increase of ECD. The poorest channel is found to be P7 channel (*Per = u*(*x, P7, α*)) because no subject shows increasing *α* power percentage there as the ECD increases.

## 4. ECD Prediction Model

Based on the experimental results of proof-of-concept study and the driving simulation study, the linear relationship between ECD and occipital EEG, particularly the *α* power percentage at O2 channel, are proved. To further estimate the ECD using the best EEG feature (*Per = u(x, O*2, *α)*), three regression models are adopted in this paper: (1) linear SVR, (2) non-linear SVR with RBF kernel, (3) non-linear SVR with Poly kernel. For evaluating the regression models using the 20 subjects in the real-life application (driving experiment), leave one subject out mean squared error (LOO-MSE), squared correlation coefficient (LOO-SCC) and estimation accuracy are calculated. Both MSE and SCC are popular and useful indexes for assessing the performance of the regression model [18,28]. The construction of SVR models was implemented by MATLAB^®^ version of well-known LibSVM^®^ (Ver. 3.17) [29].

### 4.1. SVR

The support vector regression (SVR) is a popular approach for estimating real-valued functions by constructing a decision surface that lies close to as many of the datasets as possible. It is the extension of standard support vector machine classifier that was originally designed to estimate just integer labels. With the success of LibSVM, SVR is already coding and packaged in Java, C and MATLAB language. Therefore, SVR can be easily implemented on multiple platforms, such as wearable and mobile device (e.g., Android wear device), microprocessor-based device and PC. These are the reasons for its use in this study.

SVR can be categorized into linear SVR (LSVR) and non-linear SVR (NLSVR) depending on the kernel types. A LSVR model that uses the ε-insensitive loss function can be formulated as minimization of Equation (7) as the following:

(7) $$\begin{array}{c} \min\frac{1}{2}{\left\|\vec{w}\right\|}^{2}+C\sum_{i=1}^{n}\left(\xi_{i}+\xi_{i}^{*}\right),\\ ECD_{i}-(\vec{w}\cdot f_{i}+b)\leq \varepsilon +\xi_{i},\\ \text{subject}\;\text{to}\;ECD_{i}-(\vec{w}\cdot f_{i}+b)\geq -\varepsilon -\xi_{i}^{*},\\ \xi_{i},\xi_{i}^{*}\geq 0,i=1,\ldots \ldots ,n \end{array}$$

where, the *w⃗* is a vector perpendicular to the decision surface, *b* is a scalar (decision surface bias), *ξ_i_*, $\xi_{i}^{*}$ are two slack variables for measuring the cost of errors and *C* is a user-defined parameter that denotes trade-off between the minimization problem and subjected conditions. Similarly, a NLSVR model that uses the ε-intensive loss function can be formulated as minimization of Equation (8) as follows:

(8) $$\begin{array}{c} \min\frac{1}{2}{\left\|\vec{w}\right\|}^{2}+C\sum_{i=1}^{n}\left(\xi_{i}+\xi_{i}^{*}\right),\\ ECD_{i}-(\vec{w}\cdot \Phi (f_{i})+b)\leq \varepsilon +\xi_{i},\\ \text{subject}\;\text{to}\;ECD_{i}-(\vec{w}\cdot \Phi (f_{i})+b)\geq -\varepsilon -\xi_{i}^{*},\\ \xi_{i},\xi_{i}^{*}\geq 0,i=1,\ldots \ldots ,n \end{array}$$

where, Φ(*f_i_*) is the mapping function which is used to map each input dataset from the linearly non-separable input space ℜ*^n^* to linearly separable feature space **H**. By using kernel functions (e.g., radial basis function (RBF) and polynomial kernel function (Poly)), SVR is able to do this mapping work and accomplish the non-linear regression without the need to know explicitly what the mapping function Φ(*f_i_*) is.

### 4.2. Performance Estimation

Leave one subject out approach is a cross-validation approach, where each subject serves as a test sample. The specific steps are as follows: (1) omit one subject from the training dataset pairs; (2) train the regression models using the remaining subjects; (3) test the omitted subject using the trained model in step (2) and calculate the performance indicators (MSE and SCC); (4) repeat the steps that are listed above until each subject has been omitted and tested once; (5) calculate the overall MSE and SCC. The MSE and SCC (denoted by *r^2^*) in each step are given by Equations (9) and (10):

(9) $$\text{MSE}=\frac{1}{n}\sum_{i=1}^{n}{\left(e{\text{ECD}}_{i}-{\text{ECD}}_{i}\right)}^{2}$$

(10) $$r^{2}=\frac{{\left(n\overset{n}{\underset{i=1}{\text{∑}}}eECD_{i}ECD_{i}-\overset{n}{\underset{i=1}{\text{∑}}}eECD_{i}\overset{n}{\underset{i=1}{\text{∑}}}ECD_{i}\right)}^{2}}{\left(n\overset{n}{\underset{i=1}{\text{∑}}}{\left(eECD_{i}\right)}^{2}-{\left(\overset{n}{\underset{i=1}{\text{∑}}}eECD_{i}\right)}^{2}\right)\left(n\overset{n}{\underset{i=1}{\text{∑}}}{\left(ECD_{i}\right)}^{2}-{\left(\overset{n}{\underset{i=1}{\text{∑}}}ECD_{i}\right)}^{2}\right)}$$

In addition, considering the final purpose of this study is to apply this linear relationship between EEG and ECD to the application of driver drowsiness detection (e.g., PERCLOS), the estimated accuracy on each ECD group were investigated, where the estimated accuracy (denoted by Acc) is given by Equation (11):

(11) $$\text{Acc}=\frac{\text{Number}\_\text{of}\_\text{True}\_ECD\_\text{group}}{\text{Total}\_\text{Number}\_\text{of}\_ECD\_\text{group}}\times 100\%$$

## 5. Prediction Results

### 5.1. ECD Estimation Using Regression Model

The best feature *Per = u*(*x*, *O*2, *α*) serves as a single input to estimate the ECD using three regression models. For evaluating the regression models using 20 subjects, the LOO-MSE and LOO-*r^2^*are summarized in Table 5, where the parameter “*C*”, RBF parameter “*g*” and Poly parameter “*d*” are already optimized using a simple grid search procedure with *C* = {0.01∼500} with step of 0.01, *g* = {0.01∼1} with step of 0.01 and *d* = {1∼10} with step of 1. For parameter “*ε*”, 0.01, 0.03 and 0.05 were tested before setting with a specific *ε =* 0.01.

As can be seen from Table 5, there is not much difference between the linear and non-linear model. The optimized estimation results obtained by using linear model of *C* = 16.59, non-linear RBF model of *C* = 46.62, *g* = 0.01 and Poly model of *C* = 16.59, *d* = 1 are almost the same with the same LOO-*r^2^=* 0.930. As we know, any linear model is a special case of non-linear model. Therefore, these results in Table 5 further prove that ECD and occipital *α* power percentage indeed exhibits a linear relationship. According to the principles of structural risk minimization [30], we choose the simplest model (the linear model) here for further study.

**Figure 14.**
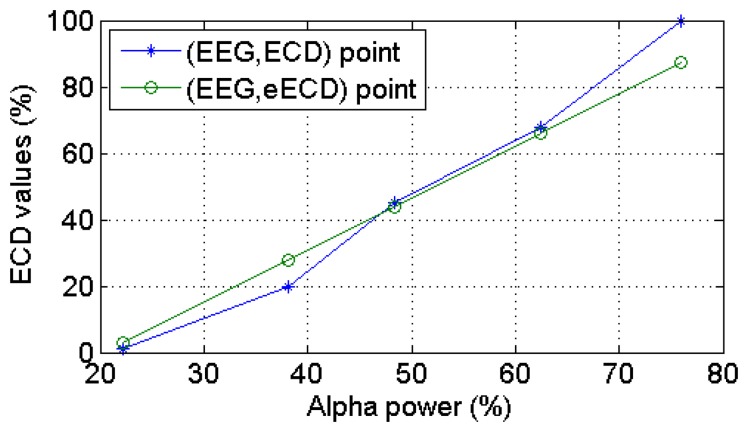
Five observed (EEG,ECD) points from subject #12 and corresponding estimated (EEG,eECD) points by using LSVR.

Table 6 shows the full ECD estimation results of the 20 subjects using the optimized LSVR model, where we can see that the best estimation results was obtained by a female subject (subject #12) with the LOO-*r^2^* = 0.987 and LOO-MSE = 0.005. Figure 14 illustrates the ECD estimations on the testing data (from subject #12) with constructed leave-subject #12-out LSVR model. The poorest estimation results was also obtained by a female subject (subject #19) with the LOO-*r^2^* = 0.78 and LOO-MSE = 0.030. Both the best and poorest estimation results are highlighted with a shaded area in Table 6.

The subjects #4, 7, 11, 13 and 15, whose results are highlighted using a bold and italic font, did not experience all five ECD groups; therefore their results are not considered for comparison with other subjects who did experience all five ECD groups. For example, we only collected two ECD segments from each of subjects #4 and #7. There is absolutely a regression line passing through the two points comprising the two EEG and ECD pairs. Thus, the outstanding *r*^2^ = 1 for subjects #4 and #7 does not make any sense.

**Figure 15.**
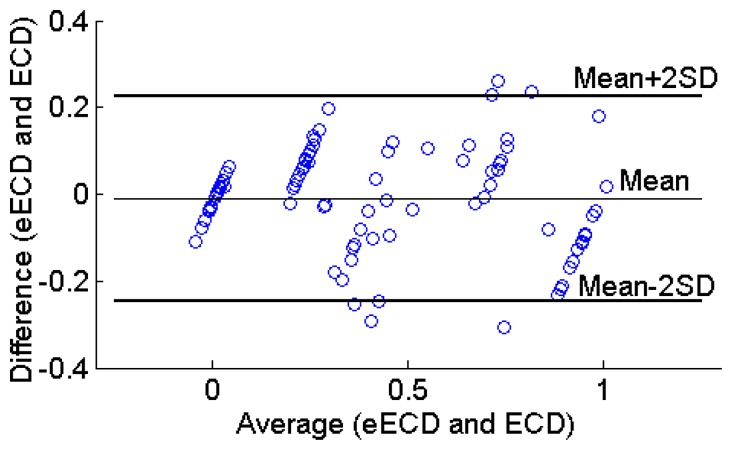
Bland-Altman plot for the relationship of the difference between the observed ECD versus estimated ECD versus their average values. Mean bias (—), +2SD, and −2SD lines are shown. SD = standard deviation.

The Bland-Altman plot is the preferred method for assessing whether an established and a new measurement technique agree [31]. It shows the paired difference between the two measurements on each subject against the mean of these two measurements. Thus, the Bland-Altman plot (Figure 15) was made for the 20 subjects. The upper and lower limits of agreement are calculated as 0.226 and −0.247, respectively. As can be seen in Figure 15, the estimated ECD values were unbiased (mean bias = 0.011) within 2SD limit (95% limits of agreement), which indicates that the estimated ECD and observed ECD values agree.

### 5.2. ECD Estimation on Different Sex

To investigate the sex difference, the mean LOO-*r^2^*and LOO-MSE values obtained by using LSVR are calculated. They are 0.928 ± 0.038 and 0.012 ± 0.006 for male subjects (except for subjects #4 and #7), and 0.909 ± 0.067 and 0.016 ± 0.004 for female subjects (except for subjects #11, #13 and #15). These results mean that male subjects have a better estimation result than female subjects. However, the independent t-test (*α* = 0.05) failed to reveal a reliable statistical difference in terms of the LOO-*r^2^* (*p-value* = 0.505) and LOO-MSE (*p-value* = 0.347) between the male and female subjects.

### 5.3. ECD Estimation on Different Daylight Condition

Drivers, particularly long-distance lorry or truck drivers, always drive in different daylight conditions, such as daytime driving and nighttime driving. Therefore, as mentioned in Section 3.2, we divided the 20 subjects into two groups comprising 10 subjects in each group for participating in daytime and nighttime driving experiments, respectively, in order to test the performance of ECD estimation in different light conditions. Experimental results show that the mean LOO-*r*^2^ and LOO-MSE are 0.948 ± 0.036 and 0.014 ± 0.009 respectively for daytime driving, which are better than the mean LOO-*r*^2^ (0.911 ± 0.062) and LOO-MSE (0.013 ± 0.009) for nighttime driving. However, the independent t-test (*α* = 0.05) failed to reveal a reliable statistical difference in terms of the LOO-*r^2^* (*p-value* = 0.120) and LOO-MSE (*p-value* = 0.847) between daytime driving and nighttime driving.

## 6. Discussion

### 6.1. ECD and Visual Cortex

The visual cortex is located in the occipital region of the brain and there is an increase in the amplitude of *α* wave if the visual input is reduced [11]. The *β* wave is associated with alertness and concentration, a good summary of its application in driver drowsiness can be found in [13]. In brief, there is a power decrease in *β* wave if the driver's alertness and concentration is decreased and there is a power increase in *β* wave if the driver's alertness and concentration is increased. In this study, with an increase of the ECD, the subjects experienced decreasing visual input and blurred the image which they could see, and as a result it is believed that their alertness and concentration were also decreased. These facts can be thought of as the reasons for the increase of RMS and *α* power and the decrease of *β* power in this study.

The fundamental difference between the previous studies and the present study is that here ECD and EEG changes are quantified. Therefore, the nature of the linear relationship between ECD and EEG can be determined. Also, previous studies did not manage to find the best occipital channel which can effectively indicate the interaction between ECD and *α* waves. In present study, a further analysis of the *α* power percentage from the four occipital channels (P7, O1, O2, P8**)** was done and channel O2 is found to be the best one. Amongst the four channels, the O1 and O2 channels are closer to the location of the visual cortex and are therefore more the widely used two positions to extract EEG features related to visual changes [32]. In addition, a previous study mentioned that the processing of visual stimuli may show right hemisphere superiority [33]. These facts can be thought of as the reason for O2's success at this time.

### 6.2. Linear EEG-ECD Relationship in Driver Drowsiness Application

PERCLOS is a video-based driver drowsiness monitoring technology. It has previously been verified that PERCLOS is the most reliable and valid in-vehicle drowsiness detection technology [5]. In order to assess whether the proposed method could be applied to compute PERCLOS, the estimation accuracy on the FC group (ECD ≥ 80%) were further investigated and compared with other four ECD groups as shown in Table 7, where we can see that FO and SC have the best two accuracies of 100% and 94.74%, followed by the FC group with an accuracy of 87.5% for male subjects and 70.0% for female subjects, while the HC group gets the lowest (38.89%). This result confirms that the proposed method is feasible for measuring PERCLOS instead of video-based method. Based on this result, the Equation (1) could be re-written as follows:

(12) $$PERCLOS=\frac{\text{Time}(LSVR(Per=u(x,O2,\alpha ))=FC)}{1\min}\times 100\%$$

**Figure 16.**
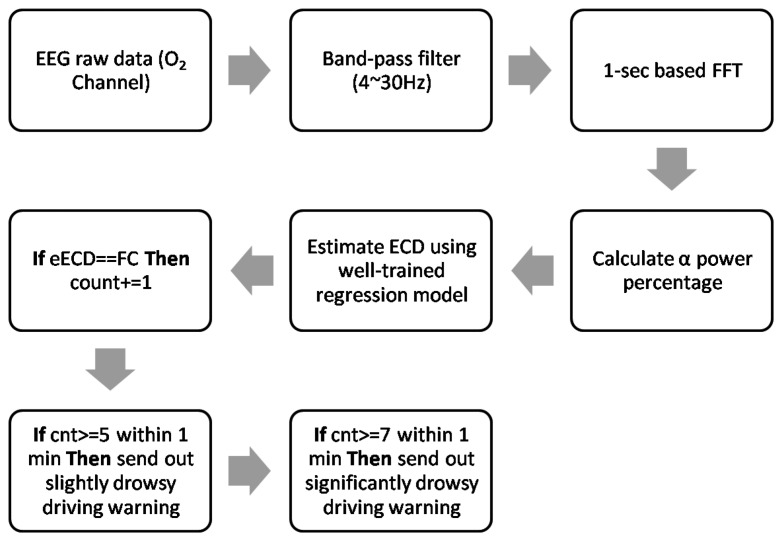
AnovelEEG-based PERCLOS algorithm.

For practical implementation, the low detection accuracy of female subjects can be easily overcome because it is common sense that most of the long-distance lorry or trunk drivers are men. This point can be indirectly verified by an Australian study [34], where the researchers recruited 1047 long-distance drivers of commercial vehicles without any special sex requirements, however almost all recruited subjects were men (99%, n = 1039). Also, it is worth to mention that the 1 s long FC duration was dominant in this study (see Table 4). Therefore, the time window is reasonably set as 1 s for extracting EEG features. In addition, according to [7], the high sensitive PERCLOS threshold is set as 8% (4.8 s) for initial advisory tone and 12% (7.2 s) for full warning, respectively. In order to get more clean EEG data, a band-pass filter with cutoff frequency from 4 Hz to 30 Hz can be used to filter out the main frequency components of eye blinks artifacts (0.5 Hz∼2 Hz [9]) and dominant energy of EMG artifacts (50 Hz∼150 Hz [35]). Thus, combining these facts, the new EEG-based PERCLOS algorithm for driver drowsiness detection can be written as Figure 16. The new ECD estimation method can be accomplished by a band-pass filter and well-trained regression model. Thus, it would be easier to implement in real time. This advance not only makes ECD and PERCLOS measurement available in nighttime driving, but it is also more computationally efficient.

**Figure 17.**
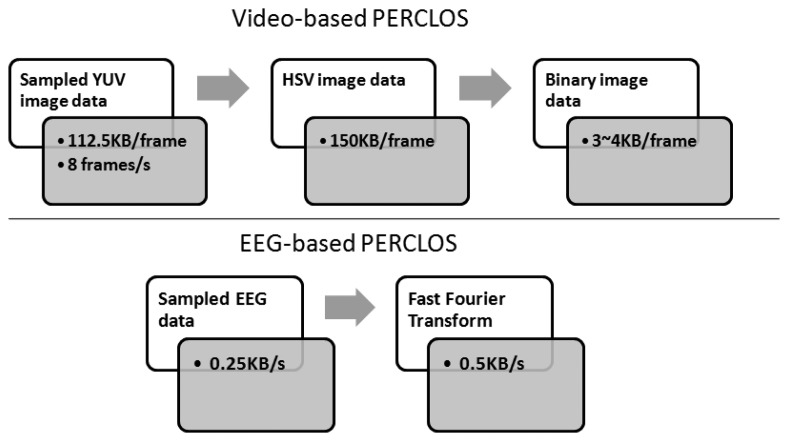
Computational load comparison between video-based (**upper**) and EEG-based PERCLOS (**bottom**).

Conventionally, the face and detection of its features are the foremost steps to extract video-based ECDs. Many researchers have presented methods to detect or track the facial features [36–41]. However, these methods cannot be practiced with fast response times for real-time applications. In our previous studies [8,14], two simplified methods that can be built based on smartphones were proposed and compared. Finally, a color-based method proved to be the most suitable one to be implemented in a smartphone device. Despite this being a major advance in in-vehicle driver drowsiness detection technology, the computational complexity is still high. To explain this further, the flowchart in Figure 17 compares the computational load of video-based and EEG-based PERCLOS methods.

In the Android smartphone platform, assuming the camera preview size and frame rate are configured as the minimum values (320 × 240 and 8 frames/s, respectively), the image data buffer is 112.5 KB for the initially default YUV format. In order to differentiate skin regions, the YUV format is then required to be encoded into the HSV color space. This needs a 150 KB memory buffer. Finally, a 3∼4 KB memory buffer is needed before converting an HSV image to a binary image (known as a black and white image) for further recognizing eyes and eye features. In contrast, an EEG-based method needs just 0.25 KB initial buffer for 1 s EEG samples (sampling rate = 128 Hz with a 16-bit ADC resolution) and twice the buffer size when running a FFT. Then the extracted FFT feature is input into the well-trained regression model for estimating ECD.

## 7. Conclusions and Future Work

In this paper, a prominent linear relationship between the ECD and the frequency domain features of the occipital EEG is found when there is not much cognitive load and when subjects are monotonously driving on a highway using a few road simulations. Following this finding, a new method using EEG sensors and linear regression is proposed for estimating the ECD instead of using video-based methods. We have shown that it is feasible to apply the proposed linear EEG-ECD model to a driver drowsiness application, PERCLOS measurement. The model-based PERCLOS not only makes PERCLOS measurements available in nighttime, but is also more computationally efficient and easier to implement in EEG sensors in a real time way.

This paper has been primarily focused on the study of the linear EEG-ECD relationship with an aim to make ECD measurement available without any brightness limitation and reducing the overall computation complexity in a long-distance monotonous driving environment where fatal drowsy driving events often happen. There are three open issues in ECD estimation using EEG which need to be further studied, such as age differences, sex differences and daylight differences. Clearly, the work presented here is not yet readily applicable to the general population of drivers and more complex driving environments.

## Figures and Tables

**Table 1.** Summary of the ECD groups' detailed information.

**Group**	**The Range of ECD Values (%)**
FO	ECD < 20
SC	20 ≤ ECD < 40
HC	40 ≤ ECD < 60
AC	60 ≤ ECD < 80
FC	80 ≤ ECD

**Table 2.** Collected observed ECD and Alpha power percentage from one subject.

**ECD Group**	**Alpha Power (%)**	**ECD (%)**
FO	40.4	0
SC	49.1	19
HC	52.1	47
AC	84.7	80
FC	85.2	100

**Table 3.** Summary of methods used in feature extraction.

	**RMS**	**SH**	**FFT**		
Domain	Time	Time	Frequency		

Measures	Mean amplitude	The amount of information	Power percentage		
			*θ*	*α*	*β*

Computational complexity	Low	Low	High		

**Table 4.** Summary of the extracted ECD segments.

**No.**	**Sex**	**Group Info**				

		**Duration (Secs); Average ECD Values(%)**				

		FO	SC	HC	AC	FC
1	M	10 ; 0	10 ; 20	2 ; 50	2 ; 70	1 ; 100
2		10 ; 1	10 ; 19	2 ; 40	10 ; 69	5 ; 100
3		10 ; 0	8 ; 20	4 ; 42	9 ; 70	2 ; 100
4		10 ; 0	10 ; 20	×	×	×
5		10 ; 0	10 ; 20	3 ; 42	3; 69	1 ; 100
6		10 ; 0	10 ; 20	1 ; 40	2 ; 60	5 ; 100
7		10 ; 2	10 ; 30	×	×	×
8		10 ; 0	10 ; 20	5 ; 43	2 ; 70	1 ; 90
9		3 ; 0	10 ; 21	10 ; 46	6 ; 70	2 ; 100
10		10 ; 0	10 ; 20	4 ; 42	10 ; 70	2 ; 100

11	F	10 ; 0	10 ; 20	10 ; 55	×	1 ; 100
12		10 ; 0	10 ; 20	10 ; 45	4 ; 68	1 ; 100
13		10 ; 0	4 ; 20	1 ; 50	×	1 ; 90
14		10 ; 0	10 ; 20	5 ; 53	2 ; 70	3 ; 90
15		10 ; 0	×	0.5 ; 40	×	1 ; 100
16		2 ; 1	10 ; 30	10 ; 43	2 ; 60	1 ; 100
17		10 ; 0	10 ; 20	3 ; 40	2 ; 70	3 ; 90
18		10 ; 0	10 ; 21	10 ; 49	1 ; 70	7 ; 100
19		10 ; 0	10 ; 20	10 ; 55	2 ; 60	1 ; 100
20		10 ; 0	10 ; 20	6 ; 42	2 ; 60	1 ; 100

**Table 5.** The optimized leave one subject out cross-validation results using linear and non-linear regression models.

**Model**		**r^2^ (SCC)**	**MSE**
LSVR	*C* = 16.59	0.930	0.013

NLSVR	*C* = 46.62	0.930	0.014
(kernel = RBF)	*g* = 0.01		

NLSVR	*C* = 16.59	0.930	0.013
(kernel = Poly)	*d* = 1		

**Table 6.** The full ECD estimation results using optimized linear and non-linear model.

**Subject**	**Sex**	**Driving Mode**	***r*^2^**	**MSE**
1			0.947	0.007
2			0.978	0.009
3		Daytime	0.931	0.010
***4***			***1.000***	***0.001***
5			0.894	0.015
	M			
6			0.888	0.022
***7***			***1.000***	***0.001***
8		Nighttime	0.874	0.017
9			0.967	0.005
10			0.942	0.007

***11***			***0.902***	***0.019***
12			0.987	0.005
***13***		Daytime	***0.937***	***0.029***
14			0.932	0.026
***15***			***0.975***	***0.017***
	F			
16			0.868	0.018
17			0.918	0.010
18		Nighttime	0.905	0.016
19			0.784	0.030
20			0.966	0.004
	Overall		0.930 ± 0.053	0.013 ± 0.009

**Table 7.** Results of Estimation accuracy for each ECD group.

**Subject**	**Sex**	**ECD Group**				

		**FO**	**SC**	**HC**	**AC**	**FC**
1	M	○	○	×	○	○
2		○	○	○	×	○
3		○	○	×	○	○
***4***		○	○	-	-	-
5		○	○	○	○	×
6		○	○	○	×	○
***7***		○	○	-	-	-
8		○	○	×	○	○
9		○	○	×	○	○
10		○	○	×	×	○

11	F	○	○	×	-	○
12		○	○	○	○	○
13		○	○	○	-	×
14		○	○	○	×	○
15		○	-	○	-	×
16		○	○	×	○	×
17		○	○	×	○	○
18		○	×	×	○	○
19		○	○	×	×	○
20		○	○	×	○	○

Overall		100%	94.74%	38.89%	66.67%	77.78%

“○”– True ECD group; “×”–False ECD group.
